# Chemical composition of solar dried blood and the ruminal content and its effect on performance of Japanese quails

**DOI:** 10.14202/vetworld.2015.82-87

**Published:** 2015-01-24

**Authors:** Jyotiprabha Mishra, Robinson J. J Abraham, V. Appa Rao, R. Asha Rajini, B. P. Mishra, N. R. Sarangi

**Affiliations:** 1 Department of Meat Science and Technology, Madras Veterinary College, Chennai, Tamil Nadu, India; 2 Department of Poultry Science, Madras Veterinary College, Chennai, Tamil Nadu, India; 3 Veterinary Dispensary, Rajsunakhala, Nayagarh, Odisha, India; 4 Department of Livestock Production Management, College of Veterinary Science & Animal Husbandry, OUAT, Bhubaneswar, Odisha, India

**Keywords:** chemical analysis, growth performance, Japanese quails, solar dried blood and rumen content

## Abstract

**Aim:**

The aim was to determine the chemical composition of solar dried blood and rumen content (DBRC) and further ascertain the concentration at which DBRC could be included in Japanese quail diets without any adverse effect on its performance.

**Materials and Methods:**

Feeding trial on the effect of DBRC on performance of Japanese quails was studied up to 5 weeks. 252 numbers of day old (Nandanam Type III breed) Japanese quails were purchased from Poultry Research Station, Madhavaram and divided into 7 batches (control+ six treatments) each consisting of 36 birds. The DBRC was included at 0%, 5%, 10%, 15%, 20%, 25% and 30% in diets as control, treatment-1 (T1), treatment-2 (T2), treatment-3 (T3), treatment-4 (T4), treatment-5 (T5) and treatment-6 (T6) respectively in a completely randomized design to replace soybean meal in Japanese quail feed. The birds were provided with *ad-labidum* feed and drinking water ad-libitum during the entire experimental period.

**Results:**

The crude protein (CP), crude fiber (CF), ether extract (EE) and ash contents of DBRC were 35.87%, 17.40%, 3.6% and 12.6%, respectively. The amount of essential amino acids and non-essential amino acid content were found to be 12.98 and 4.87 (g/100 g of feed) respectively in DBRC feed. Result showed that all birds fed DBRC diets performed better than the control group. Mortality was unaffected by dietary treatments. There was a significant difference (p<0.01) observed in weight gain in treatment groups compared to the control.

**Conclusion:**

Up to 30% DBRC could be incorporated in the diets of Japanese quails without any adverse effects on its performance.

## Introduction

It is a big challenge to make animal protein availability at an affordable cost to all sections of society because of increase in prices of different commodities. The world today is facing a huge shortage of livestock feed ingredients such as wheat, corn and soya bean etc., because of the rapid increase in human population and the competition for the feedstuff between the increased human population and livestock [1]. Feed accounts for 55-70% of the cost of poultry production. Corn and soybean meal are usually the most plentiful and well-balanced protein, thus extensively used in poultry diet. Efforts to reduce the high cost of feeds and therefore the cost of poultry products have concentrated on the use of cheaper and locally available alternative agro-by products, especially those that have no nutritional value to mankind [2]. Furthermore, the need to maximize the economic and environmental benefits in disposal of slaughterhouse by-products [3] also stimulated a renewed interest in the investigation of slaughterhouse by-products for possible use as protein feedstuffs in livestock feeds. Rumen digesta is the partially digested forage mainly found in the rumen of ruminant animals and fairly rich in crude protein (CP) (18.52%) and dried rumen digesta at 40% dietary inclusion could replace soyabean in the diet of *Oreochromis niloticus* fingerlings without compromising growth [4]. Blood meal contains about 80% CP and very rich in lysine [5] and nutritional value of blood meal increases when fed in combination with other protein sources [6]. Therefore, a combination of rumen content and blood assures a potential alternative protein source. Buffalo blood/rumen content mixture, a slaughter house by-product that offers a tremendous potential, is a cheap and locally available alternative feedstuff for livestock. Investigation had revealed the composition and potential of blood-rumen content mixture as a good source of protein in monogastric [7]. The proximate composition of bovine blood/rumen content mixture showed that it contains 45.35% CP, 4.10% ether extract (EE), 8.81% crude fiber (CF) and 15.42% ash and can replace soyabean meal up to 60% level without any deleterious effect on the carcass yield and organ weight of the finishing broilers [8]. It has been used in African countries to feed poultry [9], rabbits [10] and ruminants [11] as a cheap untraditional feedstuff to reduce feeding costs and alleviate pollution problems without any reported deleterious clinical effect on animal health and performance. The valuable taste and dietary properties of quail meat are pivotal in determining the growing interest of consumers for quail rearing. The birds are relatively easy to rear and suffer from very little maladies. The percentage content of edible meat in Japanese quail is very high [12]. However, there is limited information on the feeding value of dried blood and ruminal content for Japanese quail in India.

This study was therefore undertaken to determine the chemical composition of solar dried blood and rumen content (DBRC) and further ascertain the concentration at which DBRC could be included in Japanese quail diets without any adverse effects on its performance.

## Materials and Methods

### Ethical approval

The experiment was carried out according to the national regulations on animal welfare and Institutional Animal Ethics Committee.

### Place of work

The study has been carried out at the Department of Meat Science and Technology, Madras Veterinary College, Chennai-07 and Poultry Research Station, Madhavaram, Chennai-51.

### Feed ingredients

Rumen content was collected from 15 goat and sheep carcasses that were slaughtered at the Corporation slaughter house, Perambur, Chennai-12, shortly carcasses were weighed and eviscerated, wet rumen content was collected in colorless polyethylene bags. Later the rumen content was allowed to drain and squeezed through 0.1 mm of stainless steel sieve. Collected material was then sun dried for 7 days to ensure that end product had approximately 8-ssss9% moisture. Rumen content was collected 3 times in consecutive weeks with 1 week interval and finally pooled together. Dried rumen content was then sealed in low density polyethylene (LDPE) bags and kept in well-ventilated storage area for future use. Blood was collected from 4 beef cattle that were slaughtered at the Corporation slaughter house, Perambur, Chennai-12, and filled in polythene bag. Then the blood content was allowed to coagulate and spread over as maximum thickness of 1-2 cm on plastic sheet for sun drying to lower the moisture level approximately to 10-11%. After drying the blood, it was air tightly packed and sealed in LDPE bag and stored at room temperature for future use. The DBRC was ground and mixed in 1:3 ratios respectively and mixed at different levels in the feed of Japanese quail. Keeping in view of optimum protein content, CF content and palatability, the DBRC was selected with 1:3 ratio as reported by several researchers for preparation of feed for layer diet [13] and for rabbit [14].

### Feed formulation

The feed was formulated on the basis of protein requirement of Japanese quail according to standards prescribed by Poultry Research Station, Nandanam, Chennai-51, (22% CP for the starter ration and 20% CP for the grower ration) as presented in Tables-1-3. Control feed was prepared without DBRC and experimental feeds were prepared by incorporating DBRC in 1:3 ratios, replacing of soya bean meal on CP basis.

**Table-1:** Starter feed for Japanese quails from (0 to 3) weeks (in percent)

Ingredients	Control	T1	T2	T3	T4	T5	T6
DBRC	0	1.4	2.8	4.2	5.6	7	8.4
Maize	51.625	51.625	51.65	51.625	51.65	51.625	51.625
Bajra	0	0	0	0	0	0	0
Deoiled rice bran	4	2	2	1	1	0	0
Soyabean meal	28	26.6	25.2	23.8	22.4	21	19.6
Sunflower oil cake	0	2	2	3	3	4	4
Refined palm oil	3.5	3.5	3.5	3.5	3.5	3.5	3.5
Fish meal	10	10	10	10	10	10	10
Mineral mixture	2	2	2	2	2	2	2
Vitamin*	0.625	0.625	0.625	0.625	0.625	0.625	0.625
Salt	0.25	0.25	0.25	0.25	0.25	0.25	0.25

* Vitamin contained-(A B2 D3 K-0.01, Utracil-0.05, Unicox-0.02, Tefroli-0.05, Ultra B12-0.01, Perivac plus-0.02, Lysine-0.25, Methionine-0.20, Spectra DFM-0.01, Larvadex-0.05) parts, DBRC=Dried blood and rumen content

**Table-2:** Grower feed for Japanese quails from (3 to 5) weeks (in percent)

Ingredients	Control	T1	T2	T3	T4	T5	T6
DBRC	0	1.1	2.2	3.3	4.4	5.5	6.6
Maize	51.08	51.08	51.08	51.08	51.08	51.08	51.08
Bajra	6	6	6	6	6	6	6
Deoiled rice bran	5	4	4	3	2.5	2	1
Soyabean meal	22	20.9	19.8	18.7	17.6	16.5	15.4
Sunflower oil cake	0	1	1	2	2.5	3	4
Refined palm oil	3	3	3	3	3	3	3
Fish meal	10	10	10	10	10	10	10
Mineral mixture	2	2	2	2	2	2	2
Vitamin*	0.67	0.67	0.67	0.67	0.67	0.67	0.67
Salt	0.25	0.25	0.25	0.25	0.25	0.25	0.25

* Vitamin contained-(A B2 D3 K0.01, Utracil-0.05, Unicox-0.02, Tefroli-0.05, Ultra B12-0.01, Perivac plus-0.02, Lysine-0.25, Methionine-0.20, Spectra DFM-0.01, Larvadex-0.05) parts, DBRC=Dried blood and rumen content

**Table-3:** Proximate composition of ‘Starter and grower ration’ for Japanese quails (in percent)

Starter ration	Control	T1	T2	T3	T4	T5	T6
Nutrients							
CP	22.02	22.17	22.05	22.07	21.96	21.97	21.86
CF	3.92	4.24	4.37	4.60	4.74	4.97	5.11
EE	6.27	6.29	6.33	6.35	6.38	6.41	6.44
Lysine	1.31	1.30	1.28	1.26	1.24	1.23	1.21
Methionine	0.41	0.41	0.40	0.40	0.40	0.40	0.39
Grower ration							
CP	20.07	20.11	20.02	20.06	20.04	20.02	20.06
CF	3.79	3.99	4.09	4.29	4.45	4.60	4.80
EE	6.13	6.15	6.17	6.19	6.21	6.24	6.25
Metabolizable energy	2989.64	2989.84	2992.04	2992.24	2993.44	2994.64	2994.84
Lysine	1.17	1.16	1.14	1.13	1.23	1.10	1.09
Methionine	0.41	0.41	0.40	0.40	0.40	0.40	0.39

CP=Crude protein, CF=Crude fibre, EE=Ether extract

### Management and feeding

Two hundred fifty two numbers of day old (Nandanam Type III breed) Japanese quails were purchased from Poultry Research Station, Madhavaram and divided to 7 batches (control+ six treatments) each treatment consisted of 36 birds with 3 replication and each replication has 12 numbers of Japanese quails. All the experimental chicks were housed in well-ventilated cages with a floor space allowance of 180 cm^2^ per quail. Uniform brooding was provided for all the experimental groups. Control birds were fed with standard Japanese quail diet without dried DBRC. The next 6 treatments were provided with 5%, 10%, 15%, 20%, 25% and 30% of DBRC replacing soya bean meal in the diet. Irrespective of the treatments all the quail chicks were fed ad-libitum with the respective experimental diets and wholesome drinking water throughout the experimental period. Standard managemental practices were followed uniformly in all the groups.

The birds were reared up to 5 weeks and live weights, feed consumption of the birds were recorded at the 3^rd^ and 5^th^ week. Based on these data 3^rd^ and 5^th^ week body weight gain was calculated. The birds that died during the experiment period were subjected to routine autopsy. The age and cause of mortality were recorded.

### Proximate composition

Proximate composition *viz*., moisture, protein, fat and total ash content of feed samples were analysed by following the standard procedure [15]. Fat estimation was done in SOCS plus (Model SCS 4, Pelican Equipment Pvt. Ltd., Chennai) and protein estimation in KEL plus (Model Classic DX, Pelican Equipment Pvt. Ltd., Chennai).

### Amino acid analysis

Samples of feed (DBRC) were analyzed for amino acid content by using the instrument Agilent 1100 HP-HPLC, USA and the software used was Chemstation. Standard hydrolysis procedure [16] was followed to hydrolyze the sample with slight modifications.

### Feed Conversion Ratio (FCR)

It was determined by calculating the ratio of the total feed consumed in grams and total weight gained in grams per treatment [17].

### Statistical analysis

The data obtained in this study were analyzed statistically in SPSS software (version 20.0) as per the methods outlined by Snedecor and Cochran [18]. The significance between the treatments group were analyzed by a one-way ANOVA test.

## Results and Discussion

The proximate composition of rumen meal, blood meal and dried DBRC are presented in Table-4. The moisture, CP, EE, CF and ash content of the dried rumen content were found to be 7.36%, 18.26%, 3.6%, 24.99% and 14.47% respectively. The moisture, CP, EE, CF and ash content of the dried blood content were found to be 10.13%, 84.87%, 0.52%, 0.38% and 4.66% respectively. It was found that moisture content, CP in blood meal was highest of 10.13% and 84.87%, respectively. Whereas the rumen content had highest CF of 24.99%, ash content of 14.47% and EE of 3.6%. Whereas according to Agababiaka *et al*. [4] the moisture content was 5.47%, CP-18.58%; crude fat-3.77%; CF-34.44%; ash-18.40%. Except moisture and CF, the other nutrients were high in the present study. However, it is according to the standards prescribed by nutrient requirements of poultry [19]. The difference in the proximate composition values may be due to the differences in chemical composition of the types of pastures in which animals has grazed, period of fasting prior to slaughter and species differences [20].

**Table-4:** Proximate composition of feed (in percent)

Proximate composition (percent)	DBRC	DBC	DRC
Moisture	8.96	10.13	7.36
CP	35.87	84.87	18.26
CF	17.40	0.38	24.99
EE	3.6	0.52	3.6
Ash	12.60	4.66	14.47

DBRC=Dried blood and rumen content, DBC=Dried blood content, DRC=Dried rumen content, CP=Crude protein, CF=Crude fiber, EE=Ether extract

Further in this study the dried blood content had higher CP of 84.87% and lower moisture content of 10.13%. The CP content is more than the value reported by Togun *et al*. [10]. This difference could be due to different processing methods and the shelf life and the preservation methods followed during the analysis. However, the moisture, CF, EE and ash value are less than the values reported by Togun *et al*. [10].

The DBRC used in this study had a CP of 35.87%, moisture of 8.96%, EE of 3.6%, ash of 12.60% and a CF content of 17.40%. However, Onu *et al*. [8] reported a higher content of CP (45.35%) at replacement of soyabean at 60% which had no deleterious effect in poultry. The CF contents of 17.40% observed in the DBRC while replacing with soyabean meal in the feed is below 5% prescribed by BIS standard.

The amount of amino acid in DBRC is given in Table-5. The amount of essential amino acids and non-essential amino acid content were found to be 12.98 and 4.87 (g/100 g of feed) respectively in DBRC feed whereas requirement of essential amino acid content as per NRC [19] for Japanese quail diet 9.01 (g/100 g). Hence, the total essential amino acid content in DBRC was more than the adequate compared to NRC [19] amino acid requirements for Japanese quails from 0 to 5 weeks. The drying method is important because there is an inverse relationship between the amount of heat applied and protein digestibility. Particularly, lysine content and lysine availability decrease when the amount of heat increases, but here may be the amino acids content loss is prevented due to use of sun drying. The amount of arginine content was found to be more than the other amino acids in the DBRC and also more than the amount reported by Makinde [21]. Soybean meal has a very good amino acid balance and contains high amounts of lysine, tryptophane, threonine and isoleucine, but deficient in methionine [22]. The amount of lysine and methionine were 1.67 and 0.32 (g/100 g of feed) respectively in the feed, which were less than the values reported by Makinde [21] and amount of lysine content was more than the (1.30) and slightly less than the required methionine content of (0.50) for Japanese quail diet as per NRC [19]. This can be overcome by use of synthetic methionine reported by Makinde [23] and these results indicate the DBRC can be used as a better protein supplement for quails diet.

**Table-5:** Amino acid content (in g/100 g of feed)

Amino acids	DBRC
Essential amino acids	
Arginine	3.11
Histidine	1.17
Isoleucine	0.54
Leukine	2.25
Lysine	1.67
Methionine	0.32
Phenylalanine	1.49
Threonine	0.9
Valine	1.53
Non-essential amino acids	
Alanine	0.62
Aspartic	1.03
Glutamic	0.99
Glycine	1.00
Serine	0.76
Tyrosine	0.47

DBRC=Dried blood and rumen content

The mean of feed consumption of quails (in grams) and FCR and birds died (in per cent) of the birds in the entire period of rearing during the feeding trial are presented in Table-6. It was found that the control group had consumed the maximum feed of 949.00 g in 5 weeks, whereas treatment-6 group consumed the less amount of feed (877.13 g). In the present study, the feed consumption of quails was decreasing from the control group to treatment group. This could be due to increased appetite of the birds and the same trend was observed by Onu *et al*. [8]. The lower feed consumption of birds with inclusion of DBRC may be attributed to depressed appetite resulting from the unpleasant smell or obnoxious odor from the diets which has blood and rumen meal reported by [24]. One of the reasons for low feed consumption may also due to attainment of faster energy level due to feeding of DBRC according to [25]. In the present study the FCR of quails was decreasing from the control group to treatment group. However Japanese quails on diet DBRC appeared to have utilized the feed better than those on diets control and this will result in feed cost savings as DBRC is obtained from cheaply available rumen content [1] and blood.

**Table 6:** Mean of feed consumption of quails (in grams) and feed conversion ratio and birds died (in percent)

Treatment	0-3 week	FCR 0-3 week	3-5 week	FCR 3-5 week	Mortality % (0-5 week)
Control (0%DBRC)	328.01	3.15	621.04	7.00	13.88
T-1: (5%DBRC)	326.19	3.10	585.93	6.51	11.11
T-2: (10% DBRC)	325.22	2.88	584.86	6.35	11.11
T-3: (15% DBRC)	319.27	2.71	591.51	5.96	5.33
T-4: (20% DBRC)	314.13	2.42	596.83	6.16	11.11
T-5: (25% DBRC)	304.01	2.30	597.32	6.08	8.3
T-6: (30% DBRC)	298.60	2.07	578.53	5.78	13.88

DBRC=Dried blood and rumen content, FCR=Feed conversion ratio

The mean of the number of birds died during the entire feeding trial is presented in Table-6. The mortality rate was highest in control and treatment-6 and least in treatment-3. According to Tarasewicz *et al*. [26] in quails due to a reduction of total protein from 27.9% to 18.2%, the mortality rate was high as 11.5%. Whereas quails fed with less protein (18.2%) had the lowest mortality rate of 7.7%. In the present study, the mortality rate was lowest (5.33%) in T-3 where the feed CP was 22.07%. On the other hand, in the control that had 22.02% of CP and T-6 had a mortality rate of 13.88%. This may be due to various reasons such as pneumonic congestion, dehydration, stampeding, manage mental defects and enteritis.

The mean ± standard error of live weight of the birds are presented in Table-7 along with test of significance. The live weight of the birds on the day one from the control, T-1, T-2, T-3, T-4, T-5 and T-6 showed mean body weight (in grams) values of 9.15±0.03, 9.15±0.03, 9.16±0.02, 9.13±0.01, 9.14±0.02, 9.09±0.06 and 9.09±0.06, respectively. At the end of 5th week, birds from the control, T-1, T-2, T-3, T-4, T-5 and T-6 showed mean body weight (in grams) values of 201.60±0.25, 204.08±0.34, 213.9±0.20, 225.98±0.21, 235.69±0.33, 238.98±0.25 and 241.81±0.26, respectively. The test of significance revealed highly significant difference (p<0.01) in 5^th^ week body weight of treatments when compared to control group. Weight gains of quails (in g/head/day) are presented in Table-8. In case of body weight gain, the quails of 0 to 3 week had a body weight gain of 4.92±0.01 to 6.39±0.11 from control to T-6. Whereas the body weight gain observed from 0 to 5 week was 6.36±0.029 to 7.02±0.02. The improved performance of birds probably may be due to adequate dietary CF in the diets. Esonu *et al*. [27] had earlier reported that CF activates the intestine and more peristaltic movement, more enzyme production resulting in efficient digestion of nutrients.

**Table 7:** Mean±(SE) of body weight of quails

Treatment	Day-0	week-3	week-5
Control	9.15±0.03	112.52^a^±0.21	201.60^a^±0.25
T-1	9.15±0.03	114.12^b^±0.42	204.08^b^±0.34
T-2	9.16±0.02	121.93^c^±0.36	213.99^c^±0.20
T-3	9.13±0.01	126.8^d^±0.22	225.98^d^±0.21
T-4	9.14±0.02	138.8^e^±0.24	235.69^e^±0.33
T-5	9.09±0.06	140.88^f^±0.21	238.98^f^±0.25
T-6	9.09±0.06	143.48^g^±0.24	241.81^g^±0.26

No. of samples-30, means bearing different superscripts in the same column differ significantly. *=significant (p<0.05), **=highly significant (p<0.01), NS=Non-significant (p>0.05), SE=Standard error

**Table 8:** Weight gain of quails (in gram/head/day)

Treatment	0-3 week	3-5 week
Control (0% DBRC)	4.92^a^±0.01	6.36^a^±0.02
T-1: (5% DBRC)	4.99^b^±0.02	6.42^a^±0.03
T-2: (10% DBRC)	5.37^c^±0.01	6.57^b^±0.03
T-3: (15% DBRC)	5.60^d^±0.01	7.08^d^±0.01
T-4: (20% DBRC)	6.17^e^±0.01	6.92^c^±0.02
T-5: (25% DBRC)	6.27^f^±0.01	7.00^d^±0.02
T-6: (30% DBRC)	6.39^g^±0.11	7.02^d^±0.02

DBRC=Dried blood and rumen content

## Conclusion

Biological experiment was conducted to find out the chemical composition of solar dried DBRC and its effect on performance of Japanese quails at seven different levels of 0%, 5%, 10%, 15%, 20%, 25% and 30% with replacement of soyabean meal in starter and grower ration. The DBRC used in this study had a CP of 35.87%, moisture of 8.96%, EE of 3.6%, ash of 12.60% and a CF content of 17.40%. The amount of essential amino acids and non-essential amino acid content were found to be 12.98 and 4.87 (g/100 g of feed) respectively in DBRC feed. The results of this study indicated that the live weight of birds observed from treatment groups were higher than control group and found statistically significant. Whereas, the feed consumption of quails have decreased from the control to treatment groups. Mortality was unaffected by dietary treatments. Therefore, up to 30% DBRC could be incorporated in the diets of Japanese quails without any adverse effects on its performance.

## Authors’ Contributions

The present work was carried out during JPM’s M.V.Sc thesis program. RJJ, VAR and AR conceptualized the aim of the study, designed, planned and supervised the experiment and corrected the manuscript. Collection of samples, execution of the experimental study, collation and analysis of data, interpretation of the results and drafting the manuscript was done by JPM. BPM and NRS helped in analysis, draft and revision of the manuscript. All authors read and approved the final manuscript.
